# Subunit-dependent interfacial assembly of wheat gliadin subunits by *Lycium barbarum* polysaccharide underlies foam stabilization

**DOI:** 10.1016/j.fochx.2026.104416

**Published:** 2026-09-06

**Authors:** Chen Li, Bo Wang, Hao-yang Yu, Xing-long Dai, Tao Yang

**Affiliations:** a Shaanxi Union Research Center of University and Enterprise for Grain Processing Technologies, College of Food Science and Engineering, Northwest A & F University, Yangling 712100, China; b Public Health School, Mudanjiang Medical University, Mudanjiang 157000, China; c Wheat Improvement, Key Laboratory of Crop Ecophysiology and Farming System, Shandong agricultural university, Tai'an 271018, Shandong, China

**Keywords:** Gliadin subunits, *Lycium barbarum* polysaccharide, Foaming stability, Interfacial adsorption kinetics, Molecular dynamics simulation

## Abstract

Protein foams are governed by interfacial adsorption kinetics and film stability, yet the subunit-specific regulation of wheat gliadins by polysaccharides remains unclear. Here, the differential responses of α- and ω-gliadin to *Lycium barbarum* polysaccharide (LBP) were systematically elucidated by integrating interfacial tension kinetics, foaming performance, multiscale structure, and atomistic molecular dynamics (MD) simulations. Under the matched solvent conditions, α-gliadin exhibited higher foaming capacity and stability than ω-gliadin. LBP significantly enhanced the foam stability of both systems but through distinct mechanisms: α-gliadin exhibited β-sheet enrichment and altered aggregation behavior, accompanied by prolonged foam half-life and reduced drainage, whereas ω-gliadin showed increased conformational flexibility, surface charge, and dispersion stability, accompanied by faster apparent interfacial adsorption, prolonged foam half-life, and suppressed drainage. MD simulations further suggested that LBP was associated with a more compact and less solvent-exposed α-gliadin conformation, whereas ω-gliadin retained greater conformational variability and more transient protein–polysaccharide contacts within the sampled trajectories. Together, these results indicate subunit-dependent structural and interfacial responses of gliadin to LBP and provide molecular-level insights into the differential regulation of α- and ω-gliadin in plant protein foams.

## 1 Introduction

Food foams are thermodynamically unstable gas-liquid dispersions whose long-term stability is governed by bubble drainage, coalescence, and disproportionation (Fameau et al., 2023). With the rapid expansion of plant-based food systems, replacing traditional animal-derived foaming agents with sustainable plant proteins has become a central challenge in food colloid science (Ali et al., 2025). However, most plant proteins exhibit intrinsically weak interfacial films, resulting in rapid foam collapse and poor shelf stability. Recent studies have demonstrated that foam stability is jointly controlled by interfacial viscoelasticity and bulk-phase drainage resistance, rather than surface tension alone (Dehghani et al., 2024; Langevin, 2023), highlighting the necessity of regulating both interfacial architecture and molecular interactions at the air-water interface.

Coupling proteins with polysaccharides to form non-covalent complexes or Pickering-type stabilizers has emerged as one of the most effective strategies to enhance foam stability (Cheng et al., 2025). Polysaccharides can regulate interfacial adsorption kinetics, steric hindrance, electrostatic repulsion, and interfacial film elasticity, thereby suppressing bubble coalescence and disproportionation. For instance, Tremella polysaccharide has been shown to dramatically prolong the half-life of chickpea protein foams by inhibiting drainage and bubble coarsening (Z Wang et al., 2026). Similar stabilization mechanisms have also been reported for rice bran protein-alginate (Zheng et al., 2023), protein-pectin (Schmidt et al., 2010), and protein-soluble soybean polysaccharide systems (Li et al., 2025). These studies collectively confirm that polysaccharide-protein interactions at the interface represent a central molecular lever for foam stabilization.

Despite extensive studies on protein-polysaccharide foams at the bulk-protein level, the subunit-specific response of wheat gliadin fractions to polysaccharide regulation remains largely unexplored. Wheat gliadins are heterogeneous protein families composed of α-, β-, γ-, and ω-gliadins, which differ markedly in molecular weight, cysteine distribution, hydrophobicity, and product making quality (Chaudhary et al., 2022). Among them, α-gliadin contains intramolecular disulfide bonds and relatively compact secondary structures (Urade et al., 2018), whereas ω-gliadin lacks cysteine residues and exhibits an extended, highly surface-active conformation (Anderson et al., 2009). These intrinsic structural disparities are expected to govern their distinct adsorption kinetics, protein–polysaccharide association and interaction patterns, and responsiveness to polysaccharide modulation. However, current studies still treat gliadin even gluten as a single averaged component (Peng et al., 2023; Zhang et al., 2025), leaving the subunit-level relationship between molecular structure, interfacial behavior, and foam stability insufficiently understood.

*Lycium barbarum* polysaccharide (LBP) is a bioactive heteropolysaccharide rich in arabinose, galactose, rhamnose, and galacturonic acid units, characterized by abundant hydroxyl and carboxyl functional groups capable of forming hydrogen bonding and electrostatic interactions with proteins (H Wang et al., 2025; Xing et al., 2024). LBP is an anionic, highly hydrated heteropolysaccharide that forms thick hydration layers around proteins while electrostatically decorating protein-coated interfaces. Such hydrated polysaccharide shells provide steric and osmotic stabilization, whereas the anionic charges modulate ζ-potential and interfacial electrostatics, as shown for WPI/LBP emulsions and protein-LBP gels (Tian et al., 2021; Xing et al., 2024). Although LBP has been widely explored for its antioxidant and immunomodulatory properties, its structure-function role in regulating gliadin interfacial assembly and foam stabilization has not yet been clarified. In particular, whether LBP differentially modulates α- and ω-gliadin at the molecular and interfacial levels remains completely unknown.

Despite increasing evidence on protein–polysaccharide interactions in foam systems, whether such regulation operates through subunit-specific molecular pathways in heterogeneous proteins such as gliadin remains unclear. In particular, it is unknown whether structurally distinct gliadin subunits respond to polysaccharide modulation through fundamentally different interfacial and conformational mechanisms. We hypothesize that LBP regulates gliadin-stabilized foams through subunit-dependent pathways, in which α-gliadin undergoes enhanced intermolecular association and structural compaction, whereas ω-gliadin exhibits changes in conformational flexibility, surface charge, and dispersion stability that may contribute to its interfacial adsorption behavior.

Therefore, this study systematically investigates the differential responses of α-gliadin and ω-gliadin to LBP in terms of molecular structure, interfacial assembly, and foam stabilization. By integrating spectroscopic analysis, interfacial tension kinetics, dynamic foaming characterization, and atomistic molecular dynamics simulations, this work aims to (i) elucidate how LBP modulates the multiscale structure of distinct gliadin subunits, (ii) clarify the subunit-dependent interfacial adsorption and foam stabilization mechanisms, and (iii) establish a structure-interaction-foam performance relationship for gliadin-polysaccharide systems. This study provides a mechanistic basis for designing targeted polysaccharide strategies to stabilize plant-protein foams at the subunit level.

## 2 Materials and methods

### 2.1 Materials and sample preparation

Gliadin subunit was isolated through a two-step fractionation procedure. In the first step, total gliadin was extracted from wheat flour according to the classical Osborne method. Briefly, wheat flour was extracted with aqueous ethanol to obtain crude gliadin, which was recovered as gliadin powder after solvent removal. In the second step, α- and ω-gliadin subunits were selectively separated from the pooled gliadin extract by stepwise precipitation using aqueous ethanol of different concentrations. Specifically, the ethanol concentration was first adjusted to 40% (*v*/v), and the solution was incubated at 4 °C for 1 h to preferentially precipitate ω-gliadin. The precipitate was collected by centrifugation, washed twice with cold 40% (*v*/v) ethanol, and freeze-dried to obtain the ω-gliadin fraction. Subsequently, the ethanol concentration of the remaining supernatant was increased to 70% (*v*/v), followed by incubation at 4 °C for 1 h to precipitate α-gliadin. The α-gliadin precipitate was collected by centrifugation, washed twice with cold 70% (*v*/v) ethanol, frozen at −20 °C, and lyophilized for further use. Because ethanol fractionation enriches particular gliadin classes according to their differential solubility rather than isolating individual sequence-homogeneous proteins, the obtained samples are hereafter referred to as the α-gliadin-enriched and ω-gliadin-enriched fractions. Their differential enrichment was evaluated from their distinct SDS-PAGE molecular-weight distributions (Fig. 5a).

*Lycium barbarum* polysaccharide (LBP, Lot No. C18143323) was purchased from Macklin Biochemical Technology Co., Ltd. (Shanghai, China). The moisture, ash and protein contents of LBP were 0.59 ± 0.04%, 6.50 ± 1.21% and 1.21 ± 0.09%, respectively. The apparent weight-average molecular weight (Mw) of LBP was 32.45 kDa. Monosaccharide composition analysis revealed that LBP was primarily composed of arabinose (47.18%), galactose (26.38%), rhamnose (8.37%), glucose (7.95%), xylose (7.91%) and glucuronic acid (2.21%). LBP powder was dispersed in deionized water to prepare a 4.0% (*w*/*v*) stock solution, followed by hydration under gentle stirring for 2 h at room temperature. α-Gliadin and ω-gliadin powders were separately dispersed in 50 mM Tris-HCl buffer (pH 7.0) containing 50% (*v*/v) 1-propanol to prepare 8.0% (*w*/*v*) protein stock solutions. For preparation of the gliadin–LBP systems, each protein stock solution was mixed with an equal volume of the LBP stock solution. Importantly, the corresponding gliadin controls were prepared by mixing each protein stock solution with an equal volume of deionized water. Therefore, all four gliadin-containing systems had an identical final solvent composition of 25% (*v*/v) 1-propanol and 25 mM Tris-HCl. The final gliadin concentration was 4.0% (*w*/*v*) in all systems, while the LBP-containing systems additionally contained 2.0% (*w*/*v*) LBP. The α-gliadin and ω-gliadin samples without LBP thus served as solvent-matched controls for their respective gliadin–LBP systems. All samples were incubated at 25 °C for 1 h and subsequently equilibrated at 4 °C overnight before analysis.

### 2.2 Determination of particle size

The particle size distribution of the dispersions was measured using a laser diffraction particle size analyzer (Mastersizer 2000, Malvern Instruments, UK). Prior to measurement, all samples were diluted with deionized water to a final concentration of 0.05% (*w*/*v*) to avoid multiple scattering effects. The measurements were performed under standard optical parameters with a particle refractive index of 1.45, an absorption index of 0.02, and a dispersant (water) refractive index of 1.33. Each sample was measured in triplicate at 25 °C, and the D50 was recorded.

### 2.3 Determination of secondary structure distribution

The secondary structure of the protein samples was analyzed by circular dichroism (CD) spectroscopy. All samples were dissolved in 0.01 M phosphate-buffered saline (PBS, pH 7.4) to obtain protein solutions with a final concentration of 0.25 mg/mL. CD spectra were recorded using a J-1700 spectropolarimeter (Jasco, Tokyo, Japan) at 25 °C over a wavelength range of 190–260 nm, with a scanning speed of 100 nm/min, a bandwidth of 1 nm, and a response time of 1 s. Each spectrum represented the average of three consecutive scans after baseline correction using the corresponding buffer. The secondary structure contents, including α-helix, β-sheet, β-turn, and random coil, were calculated from the obtained spectra using Yang's algorithm.

### 2.4 Intrinsic fluorescence spectroscopy characterization

Intrinsic fluorescence spectra were recorded using a fluorescence spectrophotometer (RF-6000, Shimadzu, Japan). Protein samples (40 mg) were dispersed in 2 mL of 0.05 M acetic acid and extracted at 25 °C for 1 h, followed by centrifugation at 10,000 *g* for 15 min. The supernatant was collected for fluorescence analysis. Fluorescence emission spectra were measured at an excitation wavelength of 280 nm over an emission range of 300–420 nm. Both excitation and emission slit widths were set to 5 nm, and the photomultiplier tube (PMT) voltage was adjusted to 400 V. All measurements were performed at room temperature. And the max intrinsic fluorescence intensity (IFI) was recorded.

### 2.5 Determination of surface hydrophobicity (H₀)

Surface hydrophobicity (H₀) was determined using 8-anilino-1-naphthalenesulfonic acid (ANS) as a fluorescent probe. Samples (40 mg) were extracted in 2 mL of 50 mM acetic acid at 25 °C for 1 h and centrifuged at 10,000 ×*g* for 15 min. The protein concentration of the supernatant was determined by the BCA method, adjusted to 0.6 mg/mL, and serially diluted. ANS solution (5 μL, 8 mM) was added to 1 mL of diluted protein solution and incubated for 20 min in the dark. Fluorescence intensity was recorded at an excitation wavelength of 390 nm and an emission wavelength of 470 nm using an RF-6000 spectrofluorometer (Shimadzu, Japan). The initial slope of fluorescence intensity versus protein concentration was taken as the H₀ value.

### 2.6 Determination of zeta potential

Zeta potential was measured using a Zetasizer Nano ZS (Malvern Instruments, UK). Samples were diluted to 1.0% (*w*/*v*), loaded into disposable polystyrene cells, and analyzed at 20 °C in zeta mode. Each measurement consisted of five runs with two cycles per run.

### 2.7 Determination of interaction forces

Interaction forces were evaluated by sequential extraction using four phosphate buffer systems (0.05 M, pH 7.0) containing: 0.05 M NaCl (P1), 0.6 M NaCl (P2), 0.6 M NaCl with 1.5 M urea (P3), and 0.6 M NaCl with 8 M urea (P4), according to a reported method (Li et al., 2025). Samples (100 mg) were suspended in 10 mL of each solution, mixed at 25 °C for 1 h, and centrifuged at 10,000 ×*g* for 20 min. The soluble protein concentration in the supernatant was determined using the BCA assay. Ionic interactions, hydrogen bonding, and hydrophobic interactions were estimated from the increases in protein solubility between P1-P2, P2-P3, and P3-P4, respectively.

### 2.8 Sodium dodecyl sulfate-polyacrylamide gel electrophoresis (SDS-PAGE)

SDS-PAGE was performed using 5% (*w*/*v*) stacking gels and 12% (*w*/*v*) separating gels according to a previously reported method with minor modifications (Z Wang et al., 2026). Samples (30 μL) were mixed with 20 μL of non-reducing sample buffer [0.1 M Tris-HCl (pH 6.8), 2% (*w*/*v*) SDS, 10% (*v*/v) glycerol, and 0.1% (*w*/*v*) bromophenol blue], followed by incubation at room temperature for 1 h. The mixtures were then boiled for 5 min and centrifuged at 10,000 ×*g* for 5 min. For reducing conditions, 5% (*v*/v) β-mercaptoethanol was added to the sample buffer. Aliquots of 10 μL were loaded per lane. Electrophoresis was carried out at 80 V for stacking and 120 V for separation. Gels were stained with 0.1% (*w*/*v*) Coomassie Brilliant Blue R-250.

### 2.9 Size-exclusion high-performance liquid chromatography (SE-HPLC)

SE-HPLC analysis was performed on an LC-20 A system (Shimadzu, Japan) equipped with a Shodex Protein KW-804 column. Protein dispersions were directly mixed with an equal volume of 0.05 M sodium phosphate buffer (pH 6.8) containing 2.0% (*w*/*v*) SDS and incubated at room temperature for 1 h to ensure complete solubilization. The mixtures were then centrifuged at 5000 ×*g* for 5 min. The obtained supernatants were filtered through a 0.45 μm membrane, and 20 μL was injected into the HPLC system. Elution was carried out using 0.05 M sodium phosphate buffer (pH 6.8) containing 0.2% (*w*/*v*) SDS at a flow rate of 0.7 mL/min and a column temperature of 30 °C for 18 min. UV detection was performed at 214 nm.

### 2.10 Foaming properties and foam microstructure

Protein dispersions (20 mL) were transferred into a 100 mL graduated cylinder and whipped at 10,000 rpm for 2 min using a high-speed homogenizer (T18 digital Ultra-Turrax, IKA, Germany). The foam volume was recorded immediately after whipping and during standing at 25 °C. Foam overrun was calculated from the relative volume increase after whipping, and foam half-life was defined as the time required for the foam volume to decrease to 50% of its initial value. Foam drainage was evaluated by transferring freshly generated foam into a 50 mL graduated glass cylinder, and the volume of drained liquid collected at the bottom was recorded after 30 min. The drainage rate was calculated as the drained liquid volume divided by the drainage time. For foam microstructure observation, a small portion of freshly prepared foam was gently placed on a glass slide and observed using an optical microscope equipped with a 4× objective. Images were captured at different time intervals (0–15 min).

### 2.11 Dynamic interfacial tension and adsorption kinetics

Dynamic interfacial tension was measured using an optical contact angle analyzer (LSA100S-T, LAUDA Scientific, Germany) based on the pendant-drop method. A 10 μL droplet was formed at the tip of a 1.65 mm stainless-steel needle, and the droplet profile was continuously recorded for 600 s at 25 °C to obtain the interfacial tension-time (γ-t) curves. The initial adsorption rate (k₁), half-adsorption time (t₁/₂), equilibrium interfacial tension (γ∞), and the fitted kinetic constant (k) were extracted from the dynamic γ-t profiles using a first-order kinetic adsorption model. k₁ and t₁/₂ primarily describe the apparent kinetics of molecular adsorption at the newly formed interface, whereas γ∞ represents the apparent equilibrium interfacial tension. These parameters characterize interfacial adsorption behavior but do not directly quantify interfacial viscoelasticity or mechanical strength.

### 2.12 Molecular dynamics (MD) simulation procedures

A simplified representative arabinogalactan model of *Lycium barbarum* polysaccharide (LBP) was constructed using the Glycan Reader & Modeler module in CHARMM-GUI. The model contained 16 monosaccharide residues, comprising 14 β-d-galactopyranose (β-D-Galp) residues and two terminal α-L-arabinofuranose (α-L-Araf) residues. The main chain consisted of 12 β-D-Galp residues connected through β-(1 → 3) glycosidic linkages. Two β-D-Galp side-chain residues were attached to the O6 positions of the galactan backbone through β-(1 → 6) linkages, and each branched Galp residue carried a terminal α-L-Araf residue through an α-(1 → 3) linkage. This structure was selected as a computationally tractable arabinogalactan motif representing the major Ara- and Gal-containing domains of LBP. The representative LBP model was parameterized using the CHARMM36 carbohydrate force field in CHARMM-GUI (Park et al., 2019). The resulting topology and coordinate files were converted into a GROMACS-readable format while retaining the original CHARMM36 atom types, partial charges, bonded parameters, and Lennard–Jones parameters. This conversion changed only the file format and did not reparameterize the carbohydrate model. Protein–LBP interactions were exclusively non-covalent, and no bonded parameters were defined between the two components. Cross-component Lennard–Jones interactions were generated using the Lorentz–Berthelot combination rule, while electrostatic interactions were calculated directly from the assigned atomic partial charges using the particle mesh Ewald method. Because the protein and carbohydrate components were described using parameters originating from different force-field families, the mixed parameterization should not be regarded as a formally revalidated unified force field. Accordingly, the simulations were used primarily to compare relative conformational and interaction tendencies between the α- and ω-gliadin systems, rather than to derive quantitative binding free energies.

Individual representative amino-acid sequences were used to construct the α- and ω-gliadin models. The α-gliadin sequence corresponded to NCBI protein accession AFB35200.1 and contained 282 amino-acid residues, whereas the ω-gliadin sequence corresponded to NCBI protein accession AAT01617.1 and contained 354 residues. Their complete amino-acid sequences are provided in Table S1. These individual sequences were used to represent the contrasting class-level structural characteristics of the two gliadin types rather than all isoforms present in the corresponding ethanol-fractionated samples. Their initial three-dimensional structures were predicted using the I-TASSER server. Model confidence was evaluated using the I-TASSER C-score, estimated TM-score, and estimated RMSD, and stereochemical quality was assessed by Ramachandran analysis. The corresponding structural validation metrics are provided in Table S2.

For complex systems, the minimized LBP molecule was initially placed at a distance of approximately 1.0 nm from the protein surface without steric overlap, followed by local relaxation. This predefined configuration was used to provide a consistent starting state for comparative simulations and was not intended to represent a pre-equilibrated binding configuration. Each system was solvated in a cubic box with a minimum distance of 1.2 nm between the solute and the box boundary, using the TIP3P water model. Counterions were added to neutralize each system, followed by the addition of 15 mM NaCl to provide a defined low-ionic-strength background for all simulations. Energy minimization was conducted using the steepest-descent algorithm until the maximum force was lower than 1000 kJ·mol^−1^·nm^−1^. Equilibration consisted of 500 ps in the NVT ensemble and 1 ns in the NPT ensemble with position restraints applied to protein and LBP heavy atoms. Temperature was maintained at 298 K using the V-rescale thermostat, and pressure was controlled at 1 bar using the Parrinello-Rahman barostat. Production MD simulations were performed for 50 ns with a 2 fs time step. The 50 ns trajectories were used to compare the principal conformational and protein–LBP interaction tendencies among the systems. Trajectory stability was evaluated collectively from RMSD, Rg, SASA, RMSF, protein–LBP distance, RDF, and hydrogen-bond profiles rather than from simulation duration or a single descriptor alone. Long-range electrostatic interactions were treated using the particle mesh Ewald (PME) method, and a cut-off distance of 1.2 nm was applied for short-range interactions. All covalent bonds involving hydrogen atoms were constrained using the LINCS algorithm. Trajectories were saved every 10 ps for subsequent analyses.

Trajectory analyses were performed using GROMACS 2019. The complete 0–50 ns trajectories were used for root-mean-square deviation (RMSD) and radius of gyration (Rg) analyses. The post-relaxation 30–50 ns interval was used for root-mean-square fluctuation (RMSF), solvent-accessible surface area (SASA), hydrogen-bond, center-of-mass (COM) distance, radial distribution function (RDF), principal component analysis (PCA), and free-energy landscape (FEL) analyses. FELs were constructed using the first two principal components, and the conformations at 50 ns were used for structural visualization. Intramolecular and intermolecular hydrogen bonds were analyzed using the GROMACS *gmx hbond* module. A hydrogen bond was identified using a donor–acceptor distance cutoff of 0.35 nm and a hydrogen–donor–acceptor angle cutoff of 30°. Intramolecular hydrogen bonds were calculated within each gliadin molecule, whereas intermolecular hydrogen bonds were calculated specifically between gliadin and the representative LBP model. Moreover, because each system was represented by a single finite trajectory, the MD analyses were used to compare the conformational tendencies sampled within the present simulation window rather than to establish fully converged thermodynamic ensembles. The probability-derived free-energy landscapes therefore describe the relative distribution of conformations sampled during the analyzed trajectories. The representative snapshots were rendered using PyMOL v2.3.0 (Schrödinger, LLC).

### 2.13 Statistical analysis

All experiments were conducted in triplicate, and results are expressed as mean ± standard deviation. Statistical significance was evaluated by one-way analysis of variance (ANOVA) followed by Duncan's multiple range test at a significance level of *P* < 0.05 using SPSS 13.0 software (SPSS Inc., Chicago, IL, USA). Figures were generated using Excel 2024 and Origin 2025 (educational version).

## 3 Results analysis

### 3.1 Particle size distribution

**Fig. 1:**
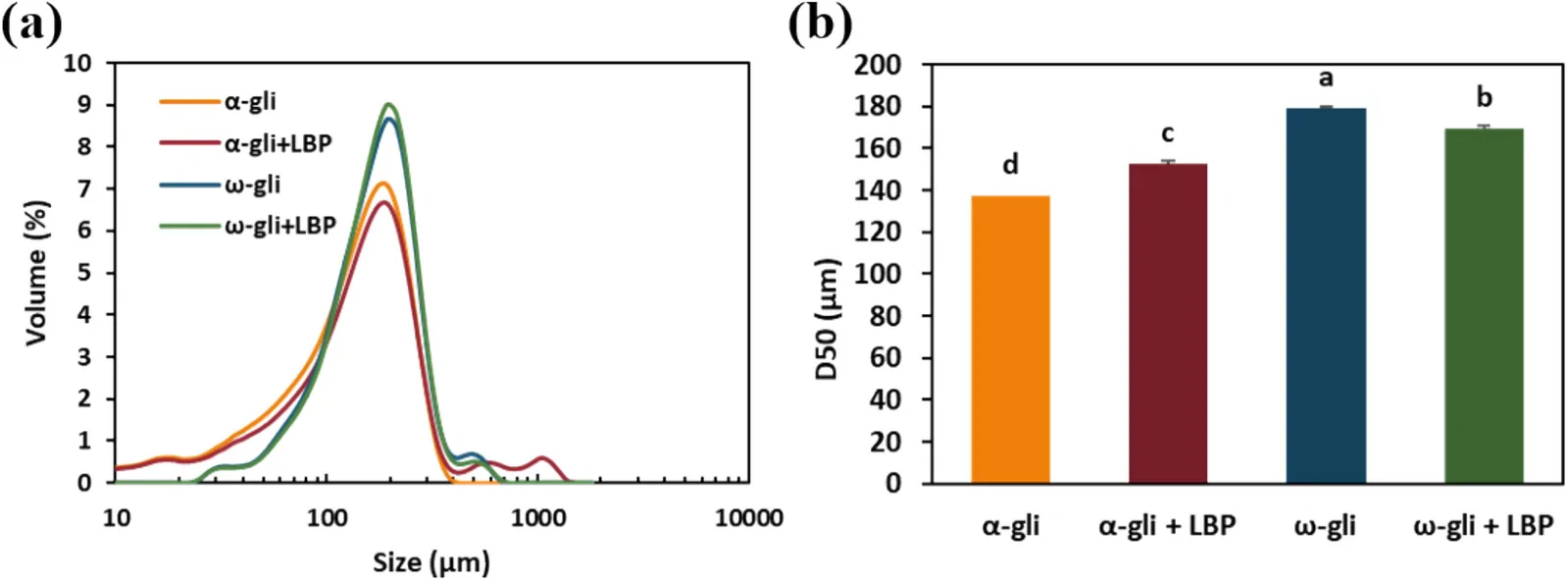
Effects of LBP on the particle size distribution of α- and ω-gliadin. (a) Particle size distribution curves and (b) corresponding D50 values.

The particle volume distribution profiles are presented in Fig. 1a, and the corresponding median particle diameters (D50) are summarized in Fig. 1b. Across all samples, the D50 values spanned from 137.47 to 179.10 μm. The smallest particle size was detected in α-gliadin (137.47 μm), whereas ω-gliadin exhibited the largest D50, reaching 179.10 μm. Upon incorporation of LBP, the two gliadin fractions showed distinct and opposite particle-size responses. For α-gliadin, the average D50 increased from 137.47 to 152.77 μm, corresponding to an 11.12% enlargement in particle size. In contrast, the addition of LBP to ω-gliadin resulted in a reduction of D50 from 179.10 to 169.60 μm, representing a 5.31% decrease.

### 3.2 Secondary structure distribution

**Fig. 2:**
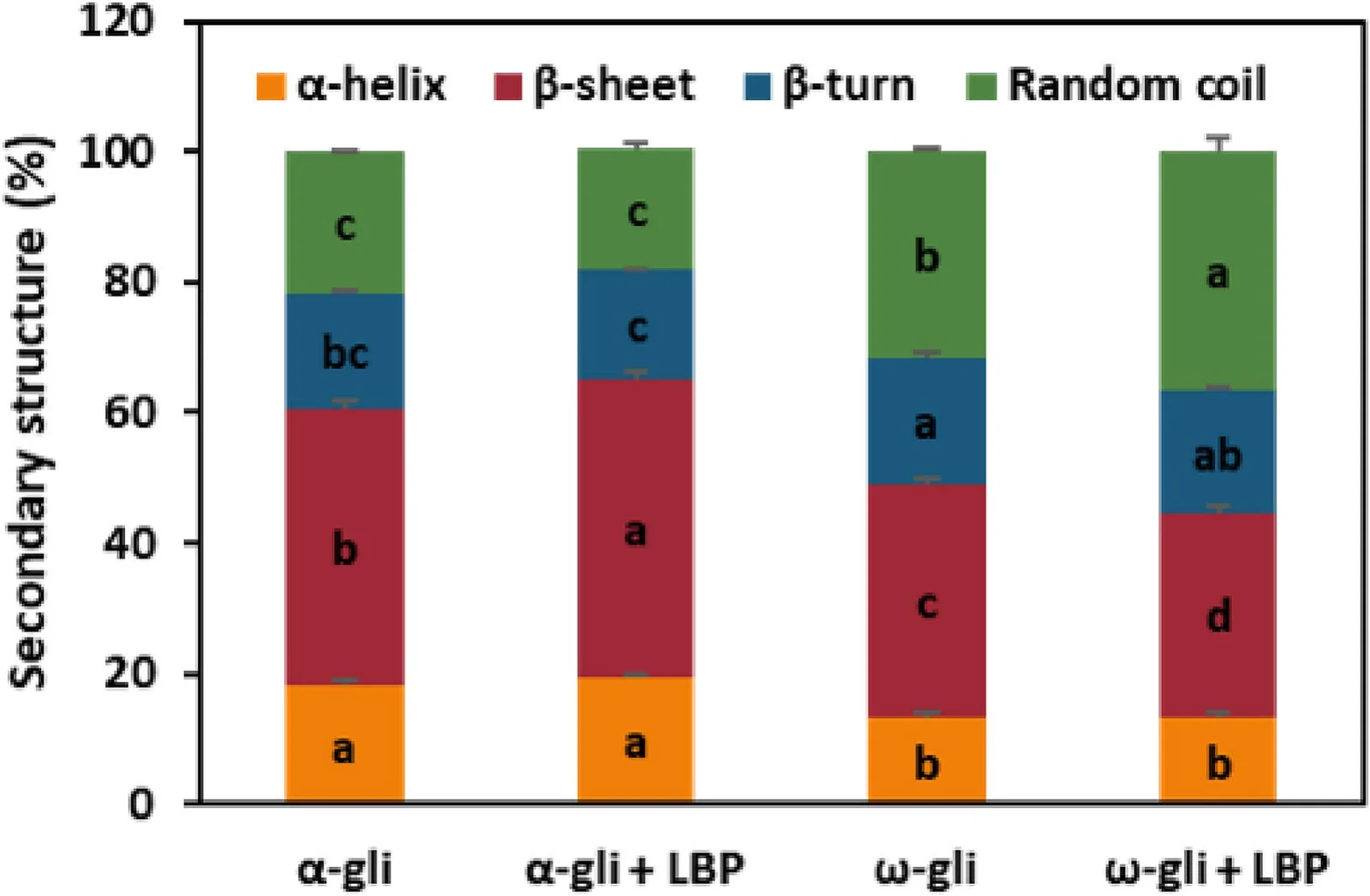
Effects of LBP on the secondary structure composition of α- and ω-gliadin.

The secondary structure distribution is presented in Fig. 2. The α-helix, β-sheet, β-turn, and random coil contents of α-gliadin were 18.37%, 42.33%, 17.60%, and 21.70%, respectively. Following LBP incorporation, the α-helix content increased to 19.47%, while the β-sheet proportion rose to 45.67%. Meanwhile, the random coil content decreased to 18.53%, representing a reduction of 14.61% relative to native α-gliadin. In contrast, ω-gliadin exhibited a lower α-helix content (13.27%) and β-sheet proportion (35.80%), accompanied by a higher random coil content of 31.43%. After LBP addition, the β-sheet proportion decreased to 31.27%, whereas the random coil content increased markedly to 36.60%, corresponding to a 16.46% increase compared with untreated ω-gliadin. The α-helix content remained essentially unchanged (13.27% to 13.40%).

### 3.3 Tertiary structure characterization

**Fig. 3:**
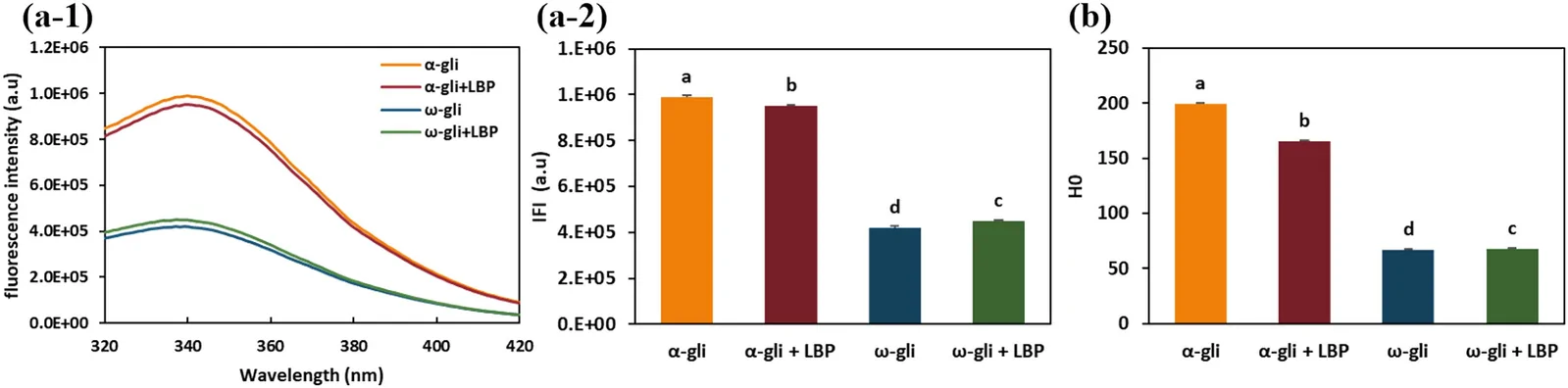
Effects of LBP on the tertiary characteristics of α- and ω-gliadin. (a-1) Intrinsic fluorescence spectra and (a-2) intrinsic fluorescence intensity (IFI) values. (b) Surface hydrophobicity (H₀).

The tertiary structural variations were evaluated using intrinsic fluorescence and surface hydrophobicity (Fig. 3a-b). Upon LBP incorporation, the intrinsic fluorescence intensity (IFI) of α-gliadin decreased by 3.89%. In contrast, the IFI of ω-gliadin was 57.35% lower than that of α-gliadin. After LBP supplementation, the IFI of ω-gliadin increased by 6.59% relative to the untreated ω-gliadin. As shown in Fig. 1d, surface hydrophobicity exhibited distinct responses to LBP addition between the two gliadin fractions. Compared with α-gliadin, the surface hydrophobicity of α-gliadin supplemented with LBP decreased by 17.18%. By contrast, ω-gliadin exhibited a much lower baseline hydrophobicity level, and its surface hydrophobicity increased slightly by 1.14% after LBP incorporation.

### 3.4 ζ-potential analysis

**Fig. 4:**
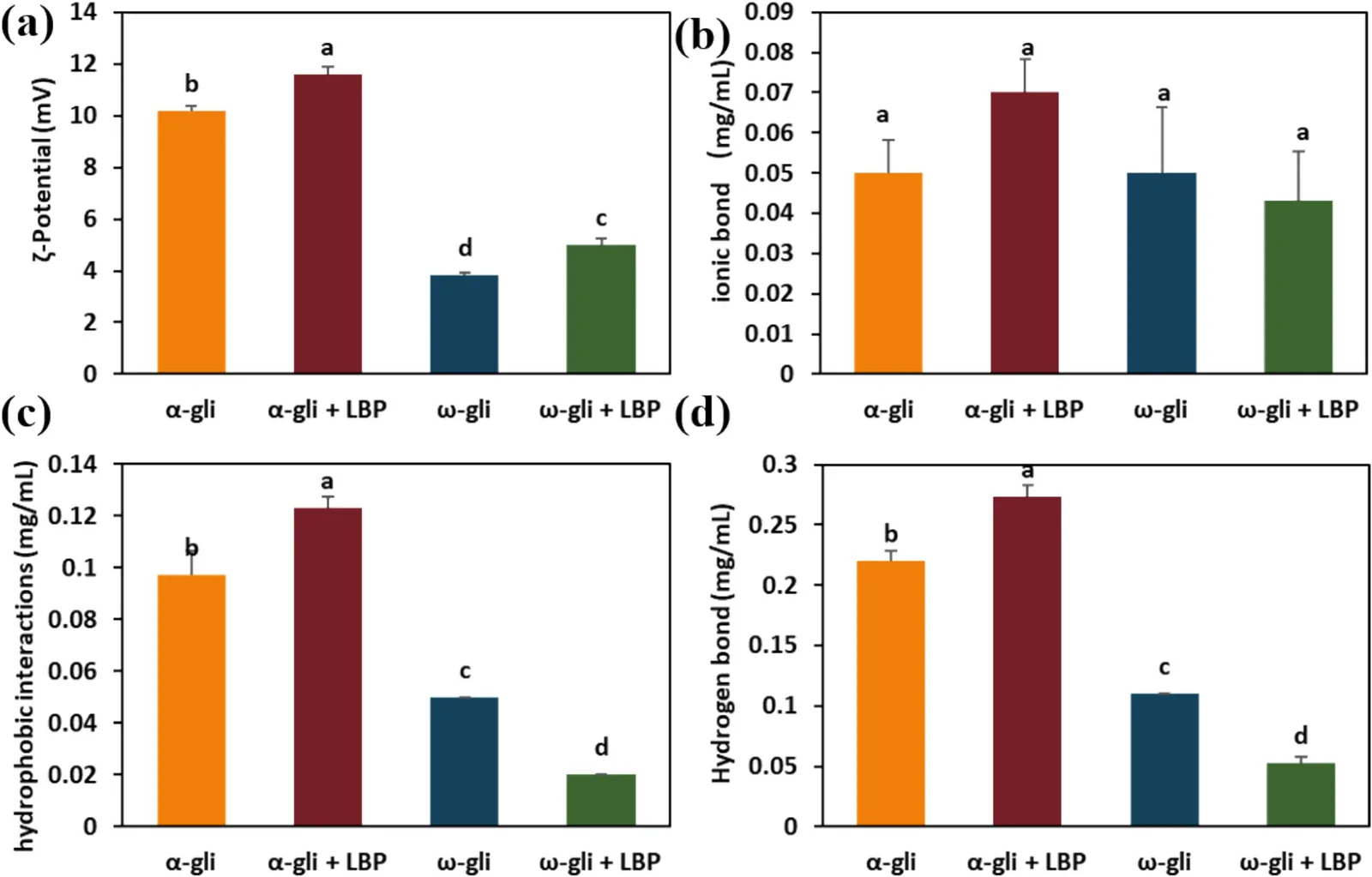
Effects of LBP on the interaction forces of α- and ω-gliadin. (a) Absolute zeta potential. (b) Ionic bonds. (c) Hydrophobic interactions. (d) Hydrogen bonds.

The ζ-potential values are presented in Fig. 4a. All samples exhibited negative ζ-potentials. The ζ-potential of the α-gliadin-enriched fraction changed from −10.14 mV to −11.60 mV after LBP addition, corresponding to a 14.38% increase in its absolute magnitude. The ω-gliadin-enriched fraction exhibited a lower absolute ζ-potential of −3.80 mV, which changed to −5.00 mV after LBP addition, representing a 31.57% increase in magnitude. Thus, LBP shifted the ζ-potentials of both fractions toward more negative values, with a larger relative change observed for the ω-gliadin-enriched fraction.

### 3.5 Interaction forces analysis

The variations in ionic bond, hydrogen bonding, and hydrophobic interactions are presented in Fig. 4b-d. For ionic bond, no significant differences were detected among the four systems, with all values remaining in a narrow range of 0.043–0.070 mg/mL, and all treatments sharing the same significance level (*p* > 0.05). In contrast, hydrogen bonding exhibited pronounced variations. Relative to the untreated α-gliadin, hydrogen bond contribution increased by 24.09% following LBP supplementation (from 0.22 to 0.273). Meanwhile, the hydrogen bonding level associated with ω-gliadin was substantially lower than that of α-gliadin, and decreased further by 51.82% after LBP incorporation (from 0.11 to 0.053). A similar but not identical trend was observed for hydrophobic interactions. Compared with α-gliadin, the hydrophobic interaction level increased by 26.80% after LBP addition (from 0.097 to 0.123). In contrast, ω-gliadin exhibited a markedly lower hydrophobic interaction level, and LBP incorporation led to a further reduction of 60.00% (from 0.05 to 0.02).

### 3.6 SDS-PAGE analysis

**Fig. 5:**
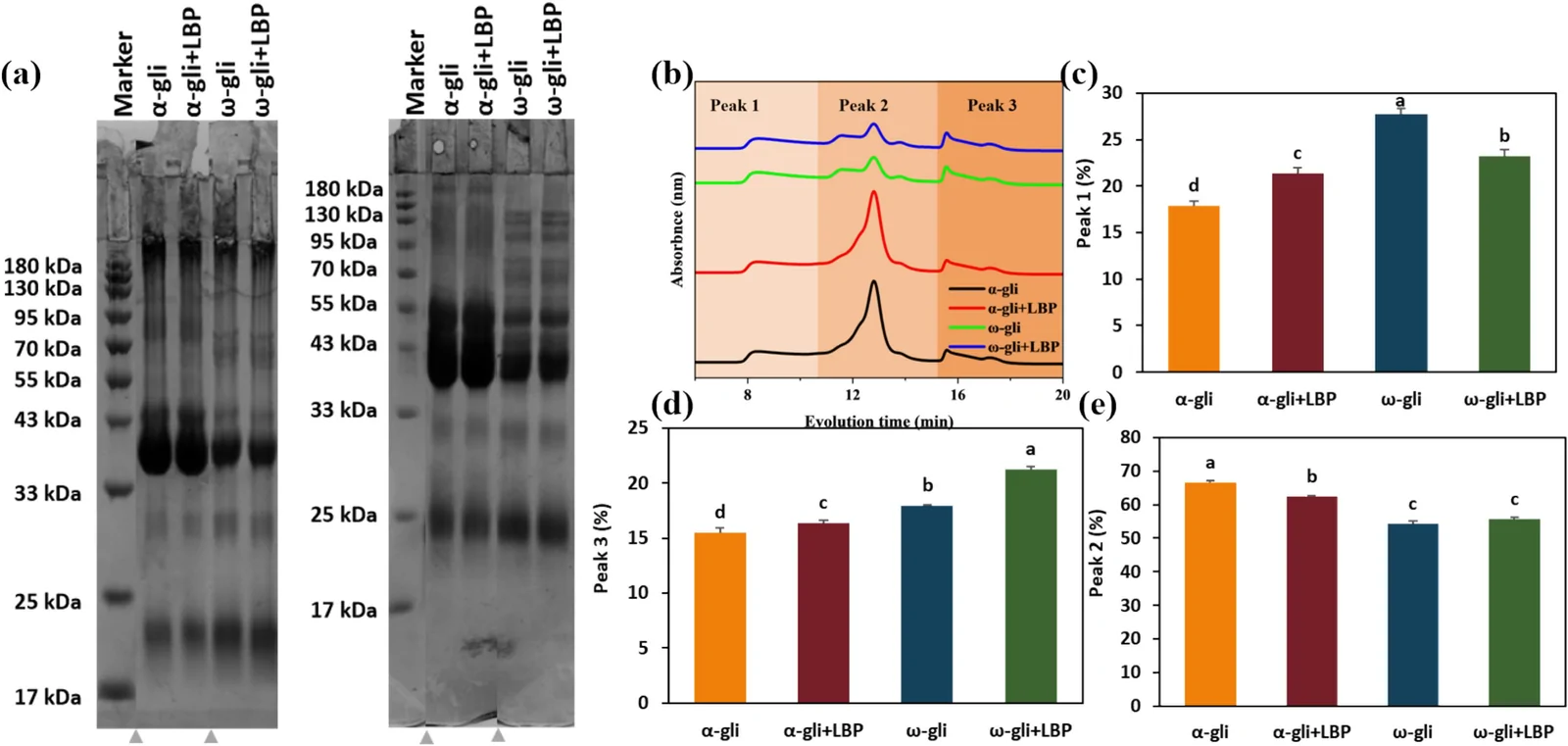
Effects of LBP on the aggregation behavior and molecular weight distribution of α- and ω-gliadin. (a) SDS-PAGE profiles under non-reducing (left) and reducing (right) conditions. All samples were run on the same SDS-PAGE gel. For figure presentation, non-adjacent lanes were repositioned while maintaining the original band intensities, and the splice boundaries were explicitly marked. (b) SE-HPLC profiles. (c-e) Relative proportions of different molecular weight fractions corresponding to Peak 1, Peak 2, and Peak 3, respectively.

Representative SDS-PAGE patterns obtained under non-reducing and reducing conditions are shown in Fig. 5a. Under non-reducing conditions, in the α-gliadin system, the high-molecular-weight region of α-gliadin supplemented with LBP showed visibly weaker staining than that of the native α-gliadin, accompanied by more diffuse residues near the top of the separating gel. For the ω-gliadin system, the opposite tendency was observed under non-reducing conditions. The ω-gliadin sample exhibited relatively faint bands in the high-molecular-weight region, whereas the corresponding bands of ω-gliadin+LBP were more clearly resolved and more intensely stained. After reduction, the banding patterns of all samples shifted toward intensified bands in the monomeric and low-molecular-weight regions. α-gliadin+LBP showed a more pronounced increase in band intensity than α-gliadin. In contrast, the monomeric and low-molecular-weight regions showed comparatively modest changes between ω-gliadin and ω-gliadin + LBP systems.

### 3.7 Molecular weight distribution analysis

The SE-HPLC profiles are shown in Fig. 5b, and the relative peak area proportions of different molecular weight fractions determined by SE-HPLC are summarized in Fig. 5c-e. Peak 1, corresponding to polymeric protein fractions, accounted for 17.83% in α-gliadin, whereas a significant increase was observed after LBP addition, reaching 21.33%. In ω-gliadin, Peak 1 exhibited a markedly higher proportion (27.70%) than that of α-gliadin. Upon LBP supplementation, the proportion of Peak 1 in ω-gliadin decreased to 23.17%. Peak 2, representing the major monomeric protein fraction, dominated the distribution in all samples. The Peak 2 proportion of α-gliadin reached 66.63%, which decreased to 62.30% after LBP incorporation. In comparison, ω-gliadin displayed a substantially lower Peak 2 proportion (54.33%). After LBP addition, a slight increase was observed, reaching 55.60%. Peak 3, corresponding to small monomeric protein fractions, exhibited the lowest proportion in α-gliadin (15.53%), which increased slightly to 16.37% following LBP treatment. In contrast, ω-gliadin showed a higher Peak 3 proportion (17.97%), and a pronounced increase was observed after LBP supplementation, reaching 21.23%.

### 3.8 Foaming properties

**Fig. 6:**
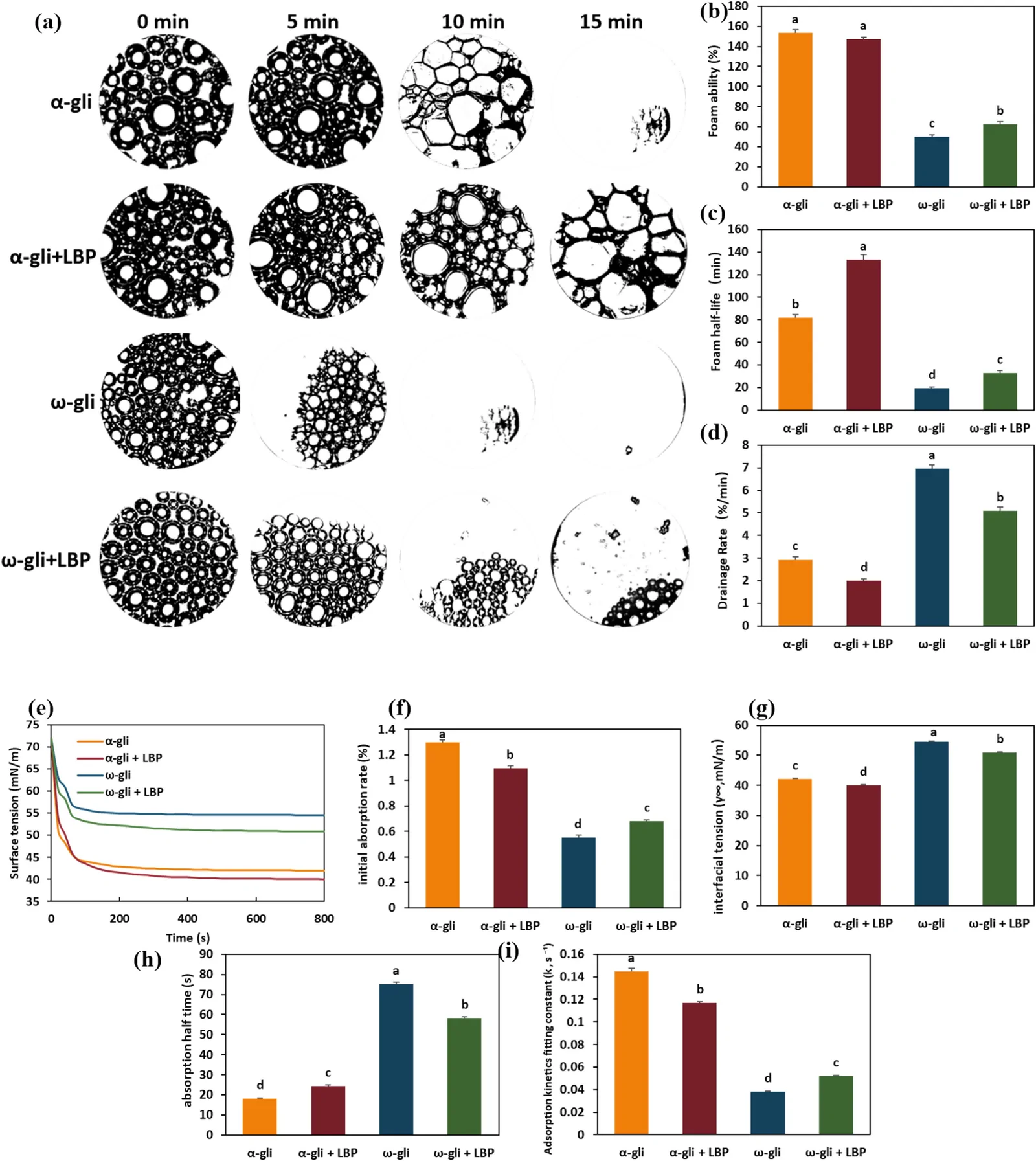
Effects of LBP on the foaming and interfacial properties of α- and ω-gliadin. (a) Optical micrographs of foams. (b) Foaming overrun. (c) Foam half-life. (d) Foam drainage rate. (e) Dynamic surface tension (γ-t) profiles. (f-i) Corresponding interfacial kinetic parameters: initial adsorption rate (k₁), half-adsorption time (t₁/₂), equilibrium interfacial tension (γ∞), and fitted kinetic constant (k). Note: All gliadin-containing samples had an identical final solvent composition of 25% (*v*/v) 1-propanol and 25 mM Tris-HCl.

The foaming performance is quantitatively evaluated using foam overrun (Fig. 6b), foam half-life (Fig. 6c), and drainage rate (Fig. 6d), and the corresponding foam microstructures are illustrated in Fig. 6a. In terms of foam overrun, α-gliadin exhibited the highest foaming capacity, reaching 153.67%, whereas ω-gliadin showed a markedly lower value of only 50.00%. Upon LBP incorporation, the foam overrun of α-gliadin decreased slightly to 147.00%, while that of ω-gliadin increased significantly to 62.33%. Compared with α-gliadin, the foam overrun of ω-gliadin was reduced by 67.47%. After LBP supplementation, the foam overrun of ω-gliadin increased by 24.67% relative to the corresponding solvent-matched gliadin control. For foam stability, as reflected by foam half-life, α-gliadin exhibited a half-life of 82 min, which was markedly higher than that of ω-gliadin (19.33 min). After LBP addition, the foam half-life of α-gliadin increased to 133 min, corresponding to an enhancement of 62.20%. Meanwhile, the foam half-life of ω-gliadin increased from 19.33 to 32.67 min after LBP supplementation, representing a 68.89% increase. Notably, the foam half-life of ω-gliadin remained substantially lower than that of α-gliadin even after LBP treatment. The drainage rate exhibited an opposite trend to the foam half-life. α-gliadin showed a relatively low drainage rate of 2.93%·min^−1^, whereas ω-gliadin displayed a significantly higher value of 6.97%·min^−1^. Upon LBP incorporation, the drainage rate of α-gliadin decreased to 2.00%·min^−1^, corresponding to a reduction of 31.80%. Similarly, the drainage rate of ω-gliadin decreased from 6.97 to 5.10%·min^−1^ after LBP treatment, with a reduction of 26.83%.

The foam microstructures are consistent with the quantitative foaming indices. The α-gliadin system exhibited a dense and uniform bubble distribution with relatively small and homogeneously dispersed bubbles. After LBP addition, the foam structure became more compact, with improved uniformity and reduced bubble coalescence. In contrast, ω-gliadin formed large, irregular, and sparsely distributed bubbles, accompanied by pronounced bubble collapse. After LBP supplementation, the bubble distribution of ω-gliadin became visibly denser and more uniform, although large bubbles and partial collapse were still evident compared with the α-gliadin systems.

### 3.9 Interfacial properties

The time-dependent adsorption behaviors at the air-water interface are presented in Fig. 6e. All samples exhibited a rapid decrease in γ at the initial stage, followed by a gradual approach to equilibrium, indicating a typical diffusion-adsorption-rearrangement process at the interface. Notably, the γ-t profiles differed distinctly among the four systems. And the interfacial adsorption kinetics parameters, including the initial adsorption rate (k₁) (Fig. 6f), half-adsorption time (t₁/₂) (Fig. 6g), equilibrium interfacial tension (γ∞) (Fig. 6h), and the fitted kinetic constant (k) (Fig. 6i) are summarized. Consistent with the γ-t curves, the initial adsorption rate (k₁) differed significantly among samples (*p* < 0.05). The k₁ value followed the order α-gliadin > α-gliadin + LBP > ω-gliadin + LBP > ω-gliadin, with α-gliadin exhibiting the highest k₁ (1.297 mN/m·s), whereas ω-gliadin showed the lowest value (0.553 mN/m·s). Correspondingly, the half-adsorption time (t₁/₂) displayed an opposite trend, with α-gliadin reaching equilibrium most rapidly (18.17 s), while ω-gliadin required the longest time (75.10 s). The equilibrium interfacial tension (γ∞) further distinguished the extent of interfacial tension reduction among the samples. The lowest γ∞ was observed for α-gli supplemented with LBP (40.00 mN/m), whereas ω-gliadin exhibited the highest γ∞ (54.53 mN/m). The fitted adsorption kinetic constant (k) showed a consistent pattern with k₁, decreasing from 0.145 s^−1^ for α-gli to 0.038 s^−1^ for ω-gliadin.

### 3.10 MD simulation analysis of gliadins and LBP interactions

The MD simulations were designed with two main objectives: (i) to characterize the LBP-induced conformational rearrangements of gliadin molecules, and (ii) to quantify the specific noncovalent interactions governing the formation and stabilization of gliadin-LBP complexes.

#### 3.10.1 Protein conformation changes characterization

**Fig. 7:**
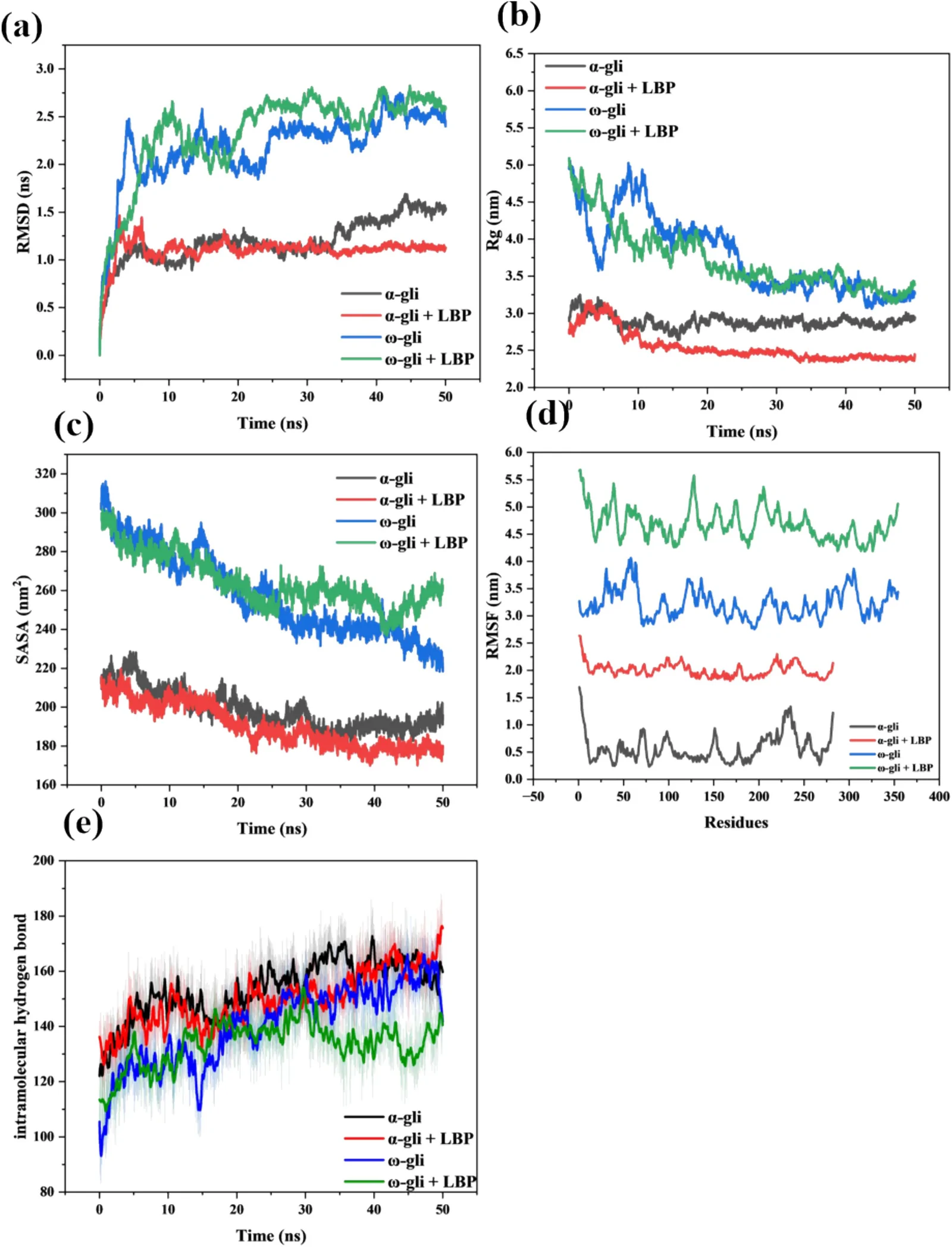
LBP-regulated conformational dynamics of α- and ω-gliadin revealed by molecular dynamics simulations. (a) Backbone root-mean-square deviation (RMSD). (b) Radius of gyration (Rg). (c) Solvent-accessible surface area (SASA). (d) Root-mean-square fluctuation (RMSF). (e) Intramolecular hydrogen bond numbers.

*RMSD analysis.* As shown in Fig. 7a, all four systems exhibited a rapid increase in RMSD within the initial equilibration stage (0–5 ns), followed by a transition to stable fluctuation regimes. For both α-gliadin systems, the RMSD stabilized at approximately 1.0–1.3 nm after 10 ns and remained relatively stable throughout the remaining simulation period. After the addition of LBP, the RMSD of α-gliadin + LBP decreased to a relatively lower plateau compared with α-gliadin alone. For ω-gliadin, the RMSD values were consistently higher than those of α-gliadin over the entire simulation trajectory. The RMSD of both ω-gliadin systems stabilized at approximately 25 ns. Specifically, the RMSD of ω-gliadin alone stabilized at around 2.3 nm, while that of ω-gliadin + LBP further increased to approximately 2.6 nm.

*Rg and SASA analysis.* As shown in Fig. 7b, the Rg of α-gliadin alone remained consistently higher (∼2.8 nm) than that of α-gliadin + LBP (∼2.2 nm) during the entire equilibrium stage. For the ω-gliadin systems, the Rg values were significantly higher than those of the α-gliadin systems over the entire simulation trajectory. The Rg of both ω-gliadin systems decreased gradually and reached stable levels after approximately 25–30 ns. Specifically, the Rg of ω-gliadin alone remained higher than that of ω-gliadin + LBP before equilibrium, while both systems converged to similar Rg values (∼3.3 nm) after equilibration. As shown in Fig. 7c, the SASA of α-gliadin + LBP (∼175 nm^2^) was consistently lower than that of α-gliadin alone (∼200 nm^2^) throughout the equilibrium stage. For the ω-gliadin systems, the SASA values were significantly higher than those of the α-gliadin systems over the entire simulation. The SASA of both ω-gliadin systems exhibited a gradual decreasing trend and reached stable levels after approximately 20 ns. During the equilibrium stage, the SASA of ω-gliadin + LBP (∼260 nm^2^) remained slightly higher than that of ω-gliadin alone (∼240 nm^2^). The relatively pronounced variations during the initial 25–30 ns, particularly for ω-gliadin, were attributed to structural relaxation from the predicted starting conformations. Thereafter, the principal structural descriptors entered comparatively stable fluctuation regimes, supporting cautious comparison of the overall conformational tendencies observed within the present trajectories.

*RMSF analysis.* As shown in Fig. 7d, α-gliadin exhibited lower RMSF values than ω-gliadin across most of the sequence. For α-gliadin, the largest fluctuations in the free form were observed at the N-terminus, particularly residues 1–6, with RMSF values up to approximately 1.7 nm. Upon LBP addition, the RMSF values of this N-terminal segment decreased markedly, resulting in a clearly reduced flexibility compared with α-gliadin alone. Pronounced RMSF reductions (|ΔRMSF| ≥ 0.30 nm) were also observed in several internal and C-terminal segments, including residues 68–73, 149–153, 225–239, 270–276, and 281–282. In contrast, ω-gliadin displayed substantially higher RMSF values than α-gliadin along most residues, with prominent peaks located in the N-terminal and middle regions. In the presence of LBP, ω-gliadin showed marked RMSF increases in multiple segments, notably residues 1–14, 37–41, 69–73, 80–86, 126–131, 171–177, 195–207, and 221–224. Conversely, several regions of ω-gliadin exhibited decreased RMSF upon LBP association, particularly residues 56–59, 63–64, which showed lower RMSF values in the ω-gliadin + LBP system compared with ω-gliadin alone.

*Intramolecular hydron bonding*. As shown in Fig. 7e, for the α-gliadin systems, the number of intramolecular hydrogen bonds increased rapidly within the first 10–15 ns and then gradually reached a stable fluctuation regime. During the equilibrium stage, the α-gliadin + LBP system exhibited a higher hydrogen bond number (∼170) than α-gliadin alone (∼150). For the ω-gliadin systems, the overall intramolecular hydrogen bond numbers were lower than those of the α-gliadin systems throughout the entire simulation. The hydrogen bond number of ω-gliadin alone increased steadily and fluctuated at a relatively high level after equilibration (∼150). Upon LBP binding, the ω-gliadin + LBP system showed a distinct decrease in the hydrogen bond number compared with ω-gliadin alone and maintained the lowest hydrogen bond level (∼130) among all four systems during the equilibrium stage.

#### 3.10.2 Protein-LBP interaction characterization

**Fig. 8:**
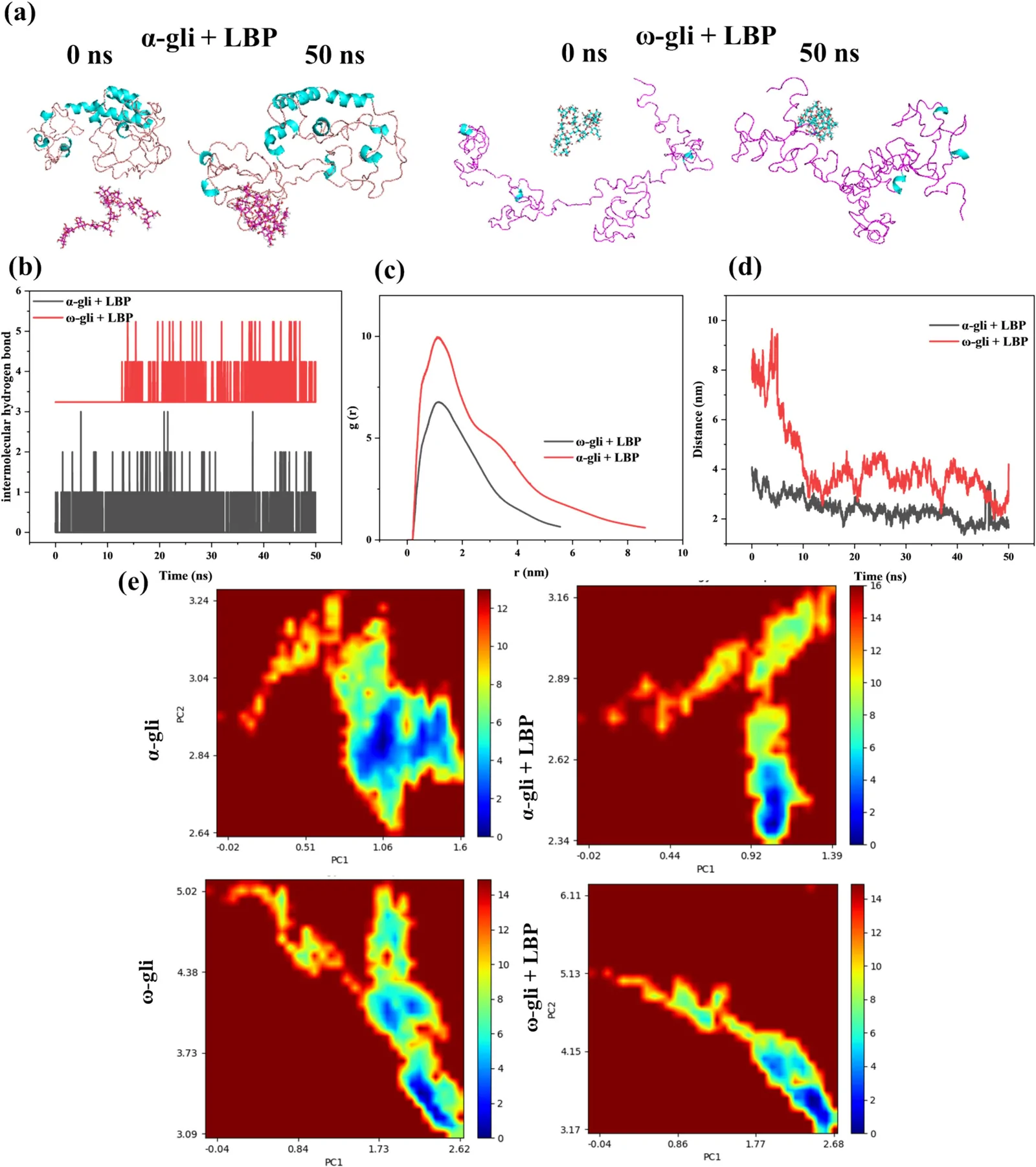
Interaction patterns and conformational distributions of α- and ω-gliadin associated with LBP revealed by molecular dynamics simulations. (a) Representative conformational snapshots at 0 and 50 ns. (b) Intermolecular hydrogen-bond numbers between gliadin and LBP. (c) Radial distribution functions between gliadin and LBP. (d) Center-of-mass distance distributions between gliadin and LBP. (e) Trajectory-derived conformational probability landscapes constructed along the first two principal components.

The representative interaction patterns between gliadins and LBP are presented in Fig. 8a. The gliadin–LBP associations were further characterized using intermolecular hydrogen bonds, radial distribution function (RDF), center-of-mass distance, and free energy landscape (FEL).

*Intermolecular hydrogen bonding.* As shown in Fig. 8b, the numbers of intermolecular hydrogen bonds formed between gliadins and LBP exhibited distinct differences between the two systems. The α-gliadin + LBP system contained a total of 773 intermolecular hydrogen bonds, whereas the ω-gliadin + LBP system exhibited a markedly lower number, with only 314 intermolecular hydrogen bonds.

*RDF analysis.* As shown in Fig. 8c, both RDF profiles exhibited a pronounced first peak at approximately 1.1 nm, and the g(r) values gradually decayed toward unity at longer distances. At around 1.10 nm, the ω-gliadin + LBP system exhibited a g(r) value of 6.77, whereas the α-gliadin + LBP system showed a higher g(r) value of 9.935 at 1.11 nm.

*Distance analysis.* As shown in Fig. 8d, for the α-gliadin + LBP system, the distance decreased rapidly during the initial simulation stage and then fluctuated around a relatively stable level throughout the remaining trajectory. The overall fluctuation amplitude remained within a relatively narrow range after equilibration. In contrast, the ω-gliadin + LBP system displayed a larger initial distance and more pronounced fluctuations during the early stage of the simulation. Although the distance gradually decreased and approached a quasi-stable regime at later stages, the fluctuation amplitude of the ω-gliadin + LBP system remained higher than that of the α-gliadin + LBP system during the sampled trajectory.

*FEL analysis.* As shown in Fig. 8f, the α-gliadin system showed a relatively broad conformational distribution along the selected principal components, whereas the α-gliadin + LBP system exhibited a more localized high-probability conformational region within the sampled trajectory. For the ω-gliadin systems, the conformational distributions showed a different spatial organization. The ω-gliadin system exhibited a wider distribution along the PC1 axis, whereas the ω-gliadin + LBP system showed a more confined high-probability region. These results indicate different conformational distributions of the two gliadin systems in the presence of LBP within the sampled simulation window.

## 4 Discussion

Foams stabilized by proteins remain thermodynamically unstable systems, and their functional stability hinges critically on the ability of proteins to rapidly adsorb, rearrange, and form a robust interfacial film at the air-water interface (Wierenga et al., 2010). In this study, α- and ω-gliadins displayed markedly different foaming behaviors where α-gliadin without LBP under the matched solvent conditions formed relatively stable foams with high overrun and long half-life, whereas ω-gliadin foams collapsed rapidly, showing low overrun and fast drainage. After LBP addition, the changes in foam formation and subsequent foam stability differed between the two gliadin fractions. For the α-gliadin-enriched fraction, LBP slightly decreased foam overrun from 153.67% to 147.00% (a reduction of 4.34%), while markedly increasing the foam half-life from 82 to 133 min and decreasing the drainage rate from 2.93 to 2.00%·min^−1^. This result reveals a trade-off between initial air incorporation and subsequent foam persistence. The slightly decreased apparent adsorption rate after LBP addition may have limited the rapid formation of new air–water interfaces during whipping, whereas the resulting protein–polysaccharide association improved resistance to drainage and bubble destabilization after foam formation. In contrast, for the ω-gliadin-enriched fraction, LBP increased foam overrun from 50.00% to 62.33% and prolonged the foam half-life from 19.33 to 32.67 min, accompanied by a decrease in drainage rate from 6.97 to 5.10%·min^−1^. This simultaneous improvement in foamability and foam stability was consistent with its increased apparent adsorption rate and shortened half-adsorption time. Therefore, LBP did not uniformly improve all aspects of foaming performance; instead, it primarily enhanced foam stability in the α-gliadin system while improving both foam formation and stability in the ω-gliadin system. The interfacial adsorption kinetics was further characterized to interpret above phenomenon. Protein-stabilized foams rely not only on equilibrium interfacial coverage, but importantly on how fast proteins reach and rearrange at the interface-because during foam generation the interface is constantly being newly formed (Fang et al., 2005). Our kinetic measurements showed that α-gliadin without LBP under the matched solvent conditions exhibits the highest initial adsorption rate (k₁) and shortest half-adsorption time (t₁/₂), consistent with rapid diffusion and anchoring at the air-water interface-explaining its relatively good foamability and stability. In contrast, native ω-gliadin had much lower k₁ and far longer t₁/₂, indicating sluggish interfacial transport and delayed film formation; this is consistent with its poor foam performance. After LBP addition, ω-gliadin's k₁ increased substantially and t₁/₂ shortened significantly, while the equilibrium interfacial tension (γ∞) decreased. This suggests that LBP increased the apparent adsorption rate of ω-gliadin, shortened its half-adsorption time, and decreased its equilibrium interfacial tension. Together with the reduced drainage rate and prolonged foam half-life, these results indicate improved interfacial adsorption and foam persistence after LBP addition. Because interfacial rheological properties were not directly measured, the enhanced foam stability cannot be specifically attributed to an increase in interfacial mechanical strength. This mechanism is consistent with studies showing that fast interfacial adsorption and high interfacial film viscoelasticity are critical for foam stabilization (Bos et al., 2001). In particular, the recent research on “protein-particle” stabilized foams indicates that rapid adsorption at the fluid interface, followed by formation of a viscoelastic interfacial network, strongly correlates with foam stability (Fameau et al., 2023). Besides, the selective and stronger impact on ω-gliadin-which had poor intrinsic interfacial behavior-indicates that LBP acts primarily via interfacial kinetic modulation and film-forming enhancement, rather than bulk rheology modification. This aligns with classical understanding that protein interfacial behavior, not bulk viscosity, governs foam stability (Damodaran, 2005). Moreover, the “compensatory enhancement” pattern observed-i.e., stronger relative improvement in the poor-performing ω-gliadin-echoes similar phenomena reported in mixed protein/polysaccharide or protein-particle foam systems (Han et al., 2023; Xu et al., 2020), where weaker interface-active proteins or particles benefit more from synergistic stabilization effects. Therefore, LBP serves as a selective interfacial performance modulator: it modestly reinforces the already efficient interfacial film of α-gliadin, but significantly reconstructs the sluggish adsorption and weak film-forming behavior of ω-gliadin. It should be noted that 1-propanol was required to maintain gliadin solubility, resulting in a final concentration of 25% (*v*/v) in all gliadin-containing samples. Because the gliadin controls and the corresponding gliadin–LBP systems were prepared with identical concentrations of gliadin, 1-propanol, and Tris-HCl, the paired differences observed after LBP incorporation cannot be attributed to variations in solvent composition. Nevertheless, 1-propanol may influence the absolute conformational and interfacial properties of gliadin. Therefore, the present results should be interpreted as the relative regulatory effects of LBP under strictly solvent-matched conditions rather than as the behavior of gliadin in an alcohol-free aqueous environment.

The subunit-dependent foam stabilization associated with LBP was accompanied by distinct changes in non-covalent interactions, protein conformation, and aggregation behavior. In protein-stabilized foams, the mechanical strength and resilience of the interfacial film depend critically on whether the adsorbed protein layer is supported by a cohesive three-dimensional aggregation network or by a flexible, weakly associated molecular assembly (Chatzigiannakis et al., 2025). Our results indicate that LBP induces distinct structural responses in α- and ω-gliadin, which are associated with their different adsorption kinetics and foam-stabilizing behaviors. First, our results of non-covalent interaction indicators‑hydrogen bonds and hydrophobic interactions-reveal that LBP addition significantly shifts the balance of inter- and intra-molecular forces. In α-gliadin + LBP, both hydrogen bonds and hydrophobic interactions increase, indicating tighter packing and stronger intermolecular cohesion; whereas in the ω-gliadin + LBP system, both interactions decrease, suggesting a more loosely associated, flexible network. This observation aligns with general principles of protein-polysaccharide (or protein-biopolymer) complexation: polysaccharides can modulate protein surface properties, induce conformational changes, and alter interaction potential via steric hindrance or electrostatic screening (Gentile, 2020; Liu et al., 2023). Such modulation of non-covalent forces directly affects the aggregation state of the proteins. Indeed, our SDS-PAGE (non-reducing) and size-exclusion chromatography (SE-HPLC) results confirm divergent aggregation behaviors: α-gliadin + LBP shows reduced high-molecular-weight bands and increased medium-size polymer peaks (Peak 1), whereas ω-gliadin + LBP shows increased presence of smaller oligomers/monomers (Peak 3) and diminished large aggregates. These shifts indicate that LBP promotes moderate aggregation of α-gliadin while maintaining a comparatively less aggregated and more flexible state in ω-gliadin. These aggregation changes were associated with the different foam-stabilization responses of the two fractions.

This kind of polysaccharide-mediated control over protein aggregation state has been documented in other food protein systems: protein-polysaccharide conjugates or complexes often exhibit lower tendency to form large insoluble aggregates, but instead adopt soluble, flexible conformations with improved interfacial activity (Nooshkam et al., 2023; Rodríguez Patino et al., 2011). For α-gliadin, LBP leads to a clear increase in β-sheet accompanied by a reduction in random coil, together with decreased intrinsic fluorescence intensity (IFI), reduced surface hydrophobicity (H₀), and a moderate increase in particle size (D50). From a structural-energetic perspective, enrichment of β-sheet structures is widely recognized as a important indicator of enhanced intermolecular hydrogen-bond cooperativity and backbone ordering (Dhami et al., 2022), which may promote more ordered protein association. β-Sheet-enriched protein assemblies have been associated with cohesive interfacial structures in other protein systems. In the present study, however, the increase in β-sheet content should be interpreted as evidence of enhanced structural ordering rather than as direct evidence of increased interfacial elasticity. The interfacial rheology studies on heat-induced protein fibrils and aggregated structures indicates that β-sheet-enriched, network-like assemblies tend to form adsorption layers with markedly higher dilatational and shear elastic moduli than their native counterparts, which in turn correlates with enhanced resistance to film thinning and rupture during drainage (Audebert et al., 2019; Jung et al., 2010). However, Zhang et al. (2025), based on correlation analysis across multiple plant proteins, reported a negative correlation between foaming capacity/stability and β-sheet content, but a positive correlation with α-helix. This difference indicates that the relationship between β-sheet enrichment and foam stability depends strongly on protein origin and aggregation state, depending strongly on protein origin and aggregation state. In contrast, the ω-gliadin + LBP system follows a completely different conformational pattern. The decrease in β-sheet content, increase in random coil proportion, and simultaneous elevation of IFI and H₀ indicate that LBP shifts ω-gliadin toward a less ordered, more solvent-exposed and dynamically flexible conformation. From the perspective of interfacial physics, an increase in conformational flexibility generally lowers the intramolecular energetic constraints that restrict protein unfolding and reorganization at newly generated interfaces. This, in turn, tends to increase the exposure and accessibility of interfacially active hydrophobic domains as well as charged residues that mediate electrostatic interactions and lateral intermolecular association. Such a “flexibility-adsorption-rearrangement” relationship has been widely observed in interfacial studies of food proteins. For instance, studies on chemically modified soy proteins and Rubisco have demonstrated that proteins with higher conformational flexibility exhibit faster interfacial adsorption, stronger interfacial tension reduction, and improved foam or emulsion stability, due to their enhanced ability to unfold and reorganize after adsorption at fluid interfaces (Lian et al., 2023; Ma, van Polen, et al., 2025). This interpretation is consistent with the pronounced increase in ζ-potential observed for the ω-gliadin + LBP system, which is expected to strengthen long-range electrostatic repulsion between protein molecules and thereby suppress irreversible bulk aggregation, maintaining a more finely dispersed and colloidally stable protein population (Huo et al., 2019). Previous studies on plant proteins and protein-polysaccharide complexes have shown that such highly charged, dispersion-stable protein species, particularly when combined with increased conformational flexibility, tend to display enhanced interfacial adsorption efficiency and improved foam or emulsion stabilization, because they can readily migrate to the interface, unfold, and reorganize to form continuous interfacial layers (Ma, Habibi, et al., 2025; YH Wang et al., 2019). In the ω-gliadin-enriched fraction, the increased conformational flexibility, surface charge, and dispersion stability were accompanied by faster apparent interfacial adsorption and improved foam persistence. These associations support a possible structure–function relationship but do not directly establish changes in interfacial mechanical properties.

Furthermore, MD simulations were used to examine the conformational stability, flexibility, hydration, and protein–LBP interaction patterns of α- and ω-gliadin. Because the initial protein structures were generated by structure prediction, relaxation from the starting conformations was expected during the early stages of the simulations. Kumar and Li (2025) demonstrated that predicted α-gliadin models may differ substantially in their initial compactness and emphasized the importance of MD-based structural relaxation before selecting and interpreting representative conformations. Consistent with this consideration, the relatively pronounced RMSD and Rg changes observed during the initial approximately 25–30 ns, particularly for ω-gliadin, were interpreted as structural relaxation rather than equilibrium fluctuations. Thereafter, the principal structural descriptors entered comparatively stable fluctuation regimes, permitting cautious comparison of the conformational and protein–LBP interaction tendencies sampled within the present 50 ns trajectories. Across all systems, the analysis of RMSD, Rg and SASA follows the standard practice in MD studies for assessing conformational stability and packing of proteins and their complexes (Sreepathi et al., 2023). Our MD simulations reveal that LBP drives α-gliadin toward a more compact and dynamically stable conformational ensemble, as evidenced by reduced RMSD fluctuations together with slight decreases in both the Rg and SASA. This behavior is consistent with the experimentally observed β-sheet enrichment and reduced random coil content, and reflects enhanced structural integrity. In contrast, ω-gliadin + LBP exhibits modest increases in Rg and SASA with slightly elevated RMSD variability, indicating a shift toward more expanded and solvent-exposed conformations rather than true destabilization. Such expansion is commonly interpreted as an increase in conformational freedom that favors functional flexibility while preserving the overall complete. In similar MD studies of myosin, whey proteins and designed proteins, environment- or ligand-induced increases in SASA and Rg have been interpreted as an expansion of conformational space that enhances functional flexibility while maintaining overall fold stability (Lu et al., 2023). Meanwhile, in ω-gliadin + LBP, RMSF increased primarily in flexible loop and tail regions, whereas the central structural motifs maintained comparable fluctuations to the native protein. This pattern suggests that LBP does not rigidify ω-gliadin; instead, it preserves the essential fold while promoting peripheral flexibility that facilitates molecular rearrangement. Similar “selective flexibilization” of loop regions has been linked in MD studies of biosurfactant proteins to improved ability to adapt to interfacial deformation and to reorganize within adsorbed layers (Cheung et al., 2018). Beyond global conformational descriptors, the interaction-level MD metrics further clarify how LBP differentially remodels α- and ω-gliadin. The RDFs of LBP around the protein surface show a higher and sharper first peak for α-gliadin + LBP, together with shorter protein-LBP center-of-mass distances and more frequent short-range contacts, indicating a more compact and persistent polysaccharide association around α-gliadin. In contrast, ω-gliadin + LBP displays a broader RDF profile, a wider distance distribution and more transient contacts, consistent with a more diffuse and dynamically exchanging polysaccharide association. These geometric differences were further reflected by the intermolecular hydrogen-bond, RDF, and center-of-mass distance analyses. Within the sampled trajectories, the representative LBP model exhibited a more localized spatial distribution and closer association with α-gliadin, whereas its association with ω-gliadin was comparatively more variable. These results indicate different modeled interaction patterns but should not be interpreted as quantitative differences in binding affinity. Such differences were reflected in the trajectory-derived conformational probability distributions obtained from the FEL analysis. Within the conformations sampled during the present trajectories, α-gliadin + LBP displayed a comparatively localized probability distribution, whereas the ω-gliadin systems occupied a broader region of the selected conformational space. These differences are consistent with lower conformational variability of α-gliadin + LBP and greater sampled heterogeneity of ω-gliadin. However, because the simulations were based on single finite trajectories, these distributions should not be interpreted as fully converged free-energy surfaces or as quantitative evidence of transition barriers between thermodynamic states.

The present MD results should be interpreted with appropriate caution. Although the principal structural descriptors entered comparatively stable fluctuation regimes after the initial relaxation period, each system was represented by a single 50 ns trajectory without independent replicas. Therefore, the simulations describe comparative conformational and interaction tendencies within the sampled trajectories rather than fully converged thermodynamic ensembles. The trajectory-derived free-energy landscapes represent only the relative conformational probabilities sampled during the simulations and cannot quantitatively determine global energy barriers or transition kinetics. In addition, the simplified arabinogalactan model does not encompass the complete structural heterogeneity of the experimental LBP sample, while the individual protein sequences used in the simulations cannot fully represent the multiple isoforms present in the ethanol-fractionated gliadin samples. The mixed AMBER99SB-ILDN/CHARMM36 parameterization has not been independently validated as a unified force field. Therefore, the simulations were used primarily to compare conformational and protein–LBP association tendencies under an identical computational protocol rather than to derive quantitative binding thermodynamics. Finally, because the simulations were performed in aqueous solution without an explicit air–water interface, the observed molecular interactions and conformational changes provide molecular-level context for the experimental observations but do not directly reproduce interfacial behavior. Longer simulations, independent replicas, representative isoform ensembles, consistently validated protein–carbohydrate force fields, and explicit-interface models will be required to further validate these findings.

## 5 Conclusion

This study indicates that *Lycium barbarum* polysaccharide (LBP) induces subunit-dependent changes in the interfacial and foaming behaviors of α- and ω-gliadin under matched solvent conditions. Experimentally, α-gliadin exhibited β-sheet enrichment and more compact association after LBP addition, accompanied by a prolonged foam half-life and reduced drainage, whereas ω-gliadin showed increased surface charge and dispersion stability together with faster apparent interfacial adsorption. The MD simulations provided complementary molecular-level indications that LBP was associated with more compact α-gliadin conformations and more persistent protein–polysaccharide contacts, whereas the ω-gliadin system sampled comparatively greater conformational variability and more transient associations within the simulated trajectories. These findings indicate distinct structural and interfacial responses of α- and ω-gliadin to LBP. However, the interfacial mechanical properties require direct verification by interfacial rheology. Future studies should employ longer simulations with independent replicas and multiple representative gliadin and LBP structures to assess the reproducibility and generalizability of the MD-derived conformational tendencies. Explicit air–water interface simulations would further help establish a more direct molecular-level relationship between gliadin–LBP interactions and interfacial or foaming behavior.

## CRediT authorship contribution statement

**Chen Li:** Writing – review & editing, Writing – original draft. **Bo Wang:** Software. **Hao-yang Yu:** Methodology. **Xing-long Dai:** Software. **Tao Yang:** Writing – review & editing, Writing – original draft, Project administration, Methodology, Funding acquisition.

## Declaration of competing interest

The authors declare that they have no known competing financial interests or personal relationships that could have appeared to influence the work reported in this paper.

## Data Availability

Data will be made available on request.

## Appendix A Supplementary data

Supplementary material A sequence and tertiary structure information of HMW-GSs.
