# Supplementary material for: Subunit-dependent interfacial assembly of wheat gliadin subunits by Lycium barbarum polysaccharide underlies foam stabilization
Source: Food Chem X. 2026 Sep 6;39:104416. doi: 10.1016/j.fochx.2026.104416 (PMC13572765; doi:10.1016/j.fochx.2026.104416)
Supplement: Supplementary file 1 [file mmc1.docx]

**Supporting information**

**Table S1.** Sequence information for the representative α- and ω-gliadin models used in the MD simulations.

| **Protein model** | **NCBI protein accession** | **Nucleotide accession** | **Cultivar** | **Length** |
| --- | --- | --- | --- | --- |
| α-gliadin | AFB35200.1 | JN831397.1 | Yumai34 | 282 aa |
| ω-gliadin | AAT01617.1 | AY591334.1 | Jinan 177 | 354 aa |

**Sequence details**

>α-gliadin

MKTFLILALLAIVATTATIAVRVPVPQLQPQNPSQQQPQEQVPLVQQQQFPGQQQPFPPQQPYPQPQPFPSQQPYLQLQPFPQPQQPYSQPQPFRPQQPYPQPQPQYSQPQQPISQQQQQQQQQQQQIIQQILQQQLIPCMDVLLQQHNIAHGRSQVLQQSTYQLLQGLCCQHPWQIPEQSQCQAIHNVVHAIILHQQQKQQQQQPSSQVSFQQPQQQYPSGQGSFQPSQQNPQAQGSLQPQQLPQFEEIRNLALQTLPAMCNVYIPPYCTIAPFGIFGTN

>ω-gliadin

MKPHHDGYKYTCSIIVTFHYPNFKHQDQKHQFQESIKHKSKMKTFIIFVLLSMPMSIVIAARHLNPSDQELQSPQQQFLEKTIISAATISTSTIFTTTTISHTPTIFPPSTTTTISPTPTTNPPTTTMTIPLATPTTTTTFSPAPTTISLATTTTISLAPTTNSPITTTTIPAATPETTTTIPPATRTNNYASTATTISLLTATTTPPATPTTILSATTTTISPAPTIISPATRTNNSLATPTTIPPATATTIPPATRTNNSPATATTIPPAPQQRFPHTRQKFPRNPNNHSLCSTHHFPAQQPFPQQPGQIIPQQPQQPLPLQPQQPFPWQPEQRSSQQPQQPFSLQPQQPFS

**Table S2.** Confidence scores and stereochemical validation of the predicted gliadin structures.

| **Gliadin model** | **I-TASSER C-score** | **Estimated TM-score** | **Estimated RMSD (Å)** | **Ramachandran favored (%)** | **Allowed (%)** | **Outliers (%)** |
| --- | --- | --- | --- | --- | --- | --- |
| α-Gliadin | −1.35 | 0.55 ± 0.15 | 8.5 ± 4.5 | 92.6 | 6.4 | 1.0 |
| ω-Gliadin | −2.10 | 0.46 ± 0.15 | 11.2 ± 4.6 | 90.8 | 7.6 | 1.6 |
